# Clinical Implication of Caloric and Video Head Impulse Tests for Patients With Enlarged Vestibular Aqueduct Presenting With Vertigo

**DOI:** 10.3389/fneur.2021.717035

**Published:** 2021-10-11

**Authors:** Ming Li, Yangming Leng, Bo Liu

**Affiliations:** Department of Otorhinolaryngology, Union Hospital, Tongji Medical College, Huazhong University of Science and Technology, Wuhan, China

**Keywords:** enlarged vestibular aqueduct, vertigo, caloric test, video head impulse test, vestibular function

## Abstract

**Background:** By examining the clinical features and results of video head impulse test (vHIT) and caloric tests in patients with enlarged vestibular aqueduct (EVA) presenting with vertigo, we aimed to investigate the function of angular vestibulo-ocular reflex (VOR) and its clinical implications.

**Methods:** Nine patients with EVA manifesting with vertigo were enrolled. The medical history, audiological examination, imaging, and the results of the caloric test and the vHIT were analyzed.

**Results:** Of the nine patients with EVA (eight bilateral and one unilateral case), five were pediatric cases. All 17 ears exhibited sensorineural hearing loss (SNHL). Enlarged vestibular aqueduct patients can present with recurrent (seven cases) or single (two cases) vertigo attack, trauma-induced (two cases), or spontaneous (seven cases) vertigo. Diminished caloric responses were observed in 77.8% (7/9) of the patients (four cases unilaterally and three bilaterally), while unilateral abnormal vHIT results in 11.1% (1/9) patients. Abnormal caloric and normal horizontal vHIT responses were found in 66.7% (6/9) of EVA patients.

**Conclusions:** Vestibular manifestations in EVA are diverse. Enlarged vestibular aqueduct patients with vertigo can present with a reduced caloric response and normal horizontal vHIT, and this pattern of angular VOR impairment was also found in other hydropic ear diseases.

## Introduction

Enlarged vestibular aqueduct (EVA) is featured by the presence of an abnormally large vestibular aqueduct generally associated with hearing loss, which was described and termed by Valvassori and Clemis in 1978 (1). The golden standard for diagnosing EVA is imaging evaluations. The initial radiographical criterion are that the diameter of the midpoint between the external aperture and the common crus of vestibular aqueduct is >1.5 mm (Valvassori criterion) (1). Most recently, more sensitive radiological criteria, the Cincinnati criteria, were put forth by defining abnormally EVAs to be >0.9 mm at the midpoint or >1.9 mm at the operculum (2). Hearing loss associated with EVA is highly variable and can be sensorineural, conductive, or mixed in nature. The clinical course can vary from fluctuating, stepwise progressive or sudden onset. Enlarged vestibular aqueduct accounts for 13–15% of sensorineural hearing loss (SNHL) in children and adolescents (3, 4).

In addition to cochlear symptoms, 4–71% of EVA patients can experience vestibular complaints, including vertigo, dizziness, head tilt, or imbalance (5). Recent advances in vestibular testing have facilitated a comprehensive evaluation of vestibular end organ function in patients with EVA and further understanding of its pathophysiological mechanisms. To date, numerous studies have addressed the otolithic dysfunction in EVA patients using vestibular evoked myogenic potentials (VEMPs), which are primarily marked by reduced thresholds and increased amplitudes (6, 7). Zhou and Gopen concluded that the abnormally augmented VEMPs response suggests a third window effect in EVA pathology and that unilateral VEMPs deficiency may be implicated in peripheral vestibular impairment (6). Moreover, the laboratory tests for evaluating angular vestibulo-ocular reflex (VOR) function mainly include the caloric test, rotation test and video head impulse test (vHIT). The caloric response reflects the VOR function of horizontal semicircular canal (SCC) at low frequency (0.002–0.004 Hz), while the newly-developed vHIT measures the VOR function of six SCCs at high frequency (5–7 Hz) (8, 9). Recently, the combination of caloric test and vHIT has provided deeper insights into the angular VOR function and the pathophysiological mechanisms of vestibular disorders (10, 11). For patients with vertigo or dizziness, the findings of the two tests are usually consistent, that is, both are normal or abnormal (10, 11). Caloric-vHIT dissociation may occur in some vestibular pathologies, mostly peripheral in origin. Caloric paresis (CP) with preserved vHIT response is usually attributed to Ménière's disease (MD) and are suggested as a vestibular indicator of endolymphatic hydrops (8, 12). While central lesions were likely to underlie those patients with abnormal vHITs and normal caloric response (11).

Until now, many previous studies have revealed unilateral or bilateral CP during caloric test in EVA patients, and this CP is attributed to dysfunction of the horizontal SCC in EVA patients (13, 14). As for vHIT application, Jung et al. (15) have investigated the performance of vHIT and caloric test in a series of EVA patients with confirmed biallelic SLC26A4 genetic mutations. Unilateral CP was found in 40% of all cases and only 25% of those with abnormal caloric responses showed abnormal vHIT results (15). In a group of pediatric EVA candidates for cochlear implantation, preoperative vestibular testing revealed normal caloric and vHIT response (16). However, in these studies, patients without vestibular symptoms were included. To our knowledge, no studies have focused on vHIT application and the relationship between caloric test and vHIT in EVA patients with vestibular complaints.

This study retrospectively analyzed the vestibular manifestations and angular VOR function of EVA patients with vertigo by using caloric test and vHIT, to explore the features and clinical significance of VOR function in EVA patients.

## Materials and Methods

### Study Populations

A single-center retrospective study was conducted in the Department of Otorhinolaryngology, Union Hospital affiliated to Tongji Medical College, Huazhong University of Science and Technology, Wuhan, China.

Nine patients with EVA presenting with vertigo were enrolled in this study. For all patients, a thorough history inquiry, otoscopy, imaging examination, and neurotological evaluations were conducted. Inclusion criteria were: (1) Enlarged vestibular aqueduct was diagnosed against the classic diagnostic criteria proposed by Valvassori and Clemis, i.e., the midpoint of the vestibular aqueduct >1.5 mm on axial CT images (1). (2) Enlarged vestibular aqueduct patients manifested with the vestibular symptoms at presentation, which were ascertained from patient/parent interview by an otolaryngologist. (3) Enlarged vestibular aqueduct patients who completed both the caloric and vHIT test.

Additionally, the exclusion criteria were: (1) other concurrent inner ear malformations; (2) middle or inner ear infections (otitis media, mastoiditis, labyrinthitis, etc.); (3) having received previous ear surgery (for example, cochlear implant) in any ear; (4) retro-cochlear lesions (vestibular schwannoma, internal acoustic canal stenosis, etc.); (5) disorders of central nervous system (vestibular migraine, multiple sclerosis, cerebellar infarction, etc.).

This study was conducted in accordance with the tenets of the Declaration of Helsinki. The study was approved by the Ethical Committee of Union Hospital Affiliated to Tongji Medical College of Huazhong University of Science and Technology, Wuhan, China.

### Audio-Vestibular Evaluations

All patients underwent audiometry, acoustic immittance, caloric test, and vHIT examination. The simple arithmetic means for frequencies of 500, 1,000, and 2,000 Hz in pure-tone audiometry is recorded as the pure tone average (PTA). The VOR function was examined on the same day during the remission period of vestibular symptoms. The Dix-Hallpike and Roll tests were also conducted. All subjects were instructed not to take alcohol, caffeine, or medications (sedative, anti-depressant drugs, etc.) that might affect the results of vestibular tests within 48 h prior to vestibular testing.

#### Bithermal Caloric Test

The infrared videonystagmography (Visual Eyes VNG, Micromedical Technologies, Chatham, IL) was performed for bithermal caloric test. The patient was placed supine with their heads lifted 30°. Each external auditory canal was alternately instilled with a constant flow of cold (24°C) and warm air (50°C) stimulation. The maximum slow phase velocity (SPV_max_) of the caloric nystagmus was recorded after each irrigation and the CP was obtained using the Jongkees formula. If the CP was ≥25%, the ear with lower response is assumed to have unilateral vestibular hypofunction, indicating an abnormal caloric reflex. In this study, bilateral vestibular hypofunction was evaluated according to the following criteria: SPV_max_ of each ear was <6°/s after caloric stimulation, or the sum of SPV_max_ was <20°/s for all four stimulation conditions (17).

#### vHIT

The ICS Impulse system (GN Otometrics, Denmark) was used for vHIT. The patient wore a pair of lightweight goggles which can record and analyze the eye movement. The patient was seated upright facing the wall at a distance of 1.0 m and was instructed to gaze at a static dot on the wall. The head were abruptly and unpredictably rotated with an amplitude of 5–15° and at a high peak velocity range of 150–200°/s. To test the horizontal SCC, the head rotations were delivered in the left or right direction. To test the vertical SCCs, patients' heads were rotated up or down in the sagittal plane with their heads turning about 45° to the left to test the right anterior-left posterior (RALP) plane or 45° to the right to test the left anterior-right posterior (LARP) plane, respectively. At least 20 head impulses were delivered in each direction. Re-fixation saccades were divided into covert and overt, and the velocity of re-fixation saccade above 50°/s was considered positive. In the present study, the appearance of a re-fixation saccades with low gain (<0.8 for the horizontal SCC and <0.7 for the vertical SCCs) was deemed pathological.

## Results

**Figure 1:**
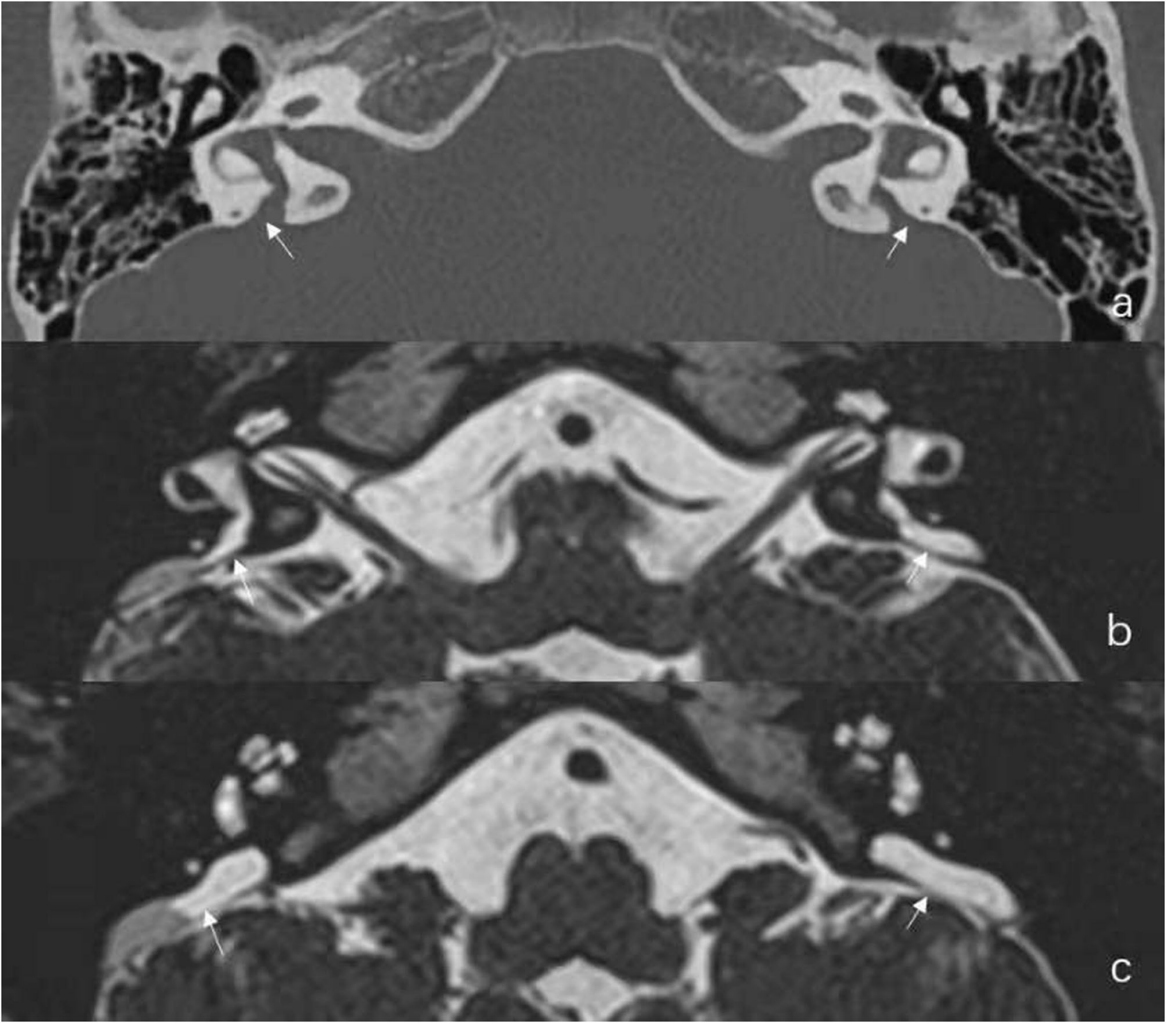
Radiological results of Case No. 2. **(a)** Axial temporal bone CT reveals the entire course of the large vestibular aqueduct extending from the crus communis of the vestibule to the posterior aspect of the petrous bone. **(b,c)** Additional axial Three-dimensional sampling perfection with application optimized contrasts using different flip angle evolutions (3D-SPACE) Magnetic Resonance Imaging (MRI) in the same child reveals enlarged endolymphatic duct (ED) and large endolymphatic sac (ES) containing variable signal intensity.

In this group, there were six females and three males, including five pediatric patients younger than 18 years old. The mean age was 16.9 years (range: 6–39 years). One case was diagnosed as unilateral EVA (case No. 1) and eight cases as bilateral EVA. Representative radiographic images are shown in Figure 1.

### Pure-Tone Audiometric and Radiological Findings

The clinical features and neurotological evaluations were shown in Table 1. All nine patients (17 ears) exhibited hearing loss. The severity of hearing loss was moderate (41–60 dB HL) in five ears (29.4%), severe (61–80 dB HL) in five ears (29.4%), and profound (>81 dB HL) in seven ears (41.2%).

**Table 1:** Clinical feature and audio-vestibular evaluations of nine patients with EVA presenting with vertigo.

**No**.	**Gender**	**Age (year)**	**Side of EVA**	**Type of hearing loss**	**Vestibular manifestation**		**PTA (dB HL)**		**Caloric test**	**vHIT gain**					
					**Causes**	**Type/duration of vertigo attack**	**R**	**L**	**CP-value (side of hypofunction)**	**R**			**L**		
										**AC**	**HC**	**PC**	**AC**	**HC**	**PC**
1	M	20	R	R: in infancy; L: none	Spontaneously	Recurrent/1 h to 1 day	103	16	19%	0.87	0.78	1.09	0.79	0.81	0.87
2	F	7	Bilat	Fluctuating and aggravation after head trauma	Head trauma	Recurrent/Few minutes	SO	51	BVH	0.73	1.03	0.86	0.77	0.96	0.71
3	F	13	Bilat	Fluctuating	Spontaneously	Recurrent/5–10 min	51	51	42% (L)	0.75	1.03	0.70	0.79	1.14	0.76
4	M	25	Bilat	Progressive	Spontaneously	Recurrent/10–30 min	78	105	13%	0.85	1.05	0.93	0.77	1.11	0.95
5	F	6	Bilat	Fluctuating and aggravation after head trauma	Head trauma	Single/one day	70	65	BVH	0.71	0.97	0.77	0.71	0.96	0.76
6	F	12	Bilat	Fluctuating	Spontaneously	Recurrent/ few minutes	56	50	33% (R)	0.81	0.98	0.99	0.83	1.02	1.11
7	M	22	Bilat	R: progressive; L: sudden aggravation	Spontaneously	Single/30 min	80	SO	74% (L)	0.70	0.98	0.74	0.23^*^	0.63^*^	0.2^*^
8	F	8	Bilat	Progressive and aggravation	Spontaneously	Recurrent/about 10 min	SO	91	26% (R)	0.71	0.94	0.9	0.89	1.01	1.01
9	F	39	Bilat	Fluctuating and aggravation	Spontaneously	Recurrent/about 1 h	76	SO	BVH	0.71	1.01	0.93	0.99	0.8	0.75

*M, male; F, female; L, left; R, right; Bilat, bilateral side; PTA, pure tone audiometry; CP, canal paresis; AC, anterior semicircular canal; HC, horizontal semicircular canal; PC, posterior semicircular canal; BVH, bilateral vestibular hypofunction; SO, scale out*.

* *Appearance of a re-fixation saccades*.

### Vestibular Manifestations

Of the nine EVA patients with vertigo, seven patients presented with spontaneous vertigo and two with vertigo induced by head trauma. In addition, seven patients exhibited recurrent episodes of vertigo and two had single episode of vertigo attack (Table 1).

An adult patient (case No. 1) with unilateral EVA presented with otherwise unexplained SNHL in childhood without a definite diagnosis. The patient presented with recurrent vertigo attacks at the age of 22 and was diagnosed with EVA in the right ear. Two pediatric EVA patients (case No. 2 and 5) presented with vertigo and imbalance following head trauma and were referred to the clinic. Five patients (case No. 3, 4, 6, 8, and 9) with EVA (two adults and three children) presented with recurrent vertigo for the past half to 3 months, with a history of bilateral hearing loss that occurred many years ago. A patient (case No. 7) presented with sudden hearing loss and vertigo in the left ear over the course of a week, who also had bilateral hearing loss for many years (more so in the right side). The duration of vertigo varied, ranging from a few minutes to a day (Table 1). No patient complained of positional vertigo, and no positional nystagmus was detected in Dix-Hallpike or Roll tests. Accordingly, benign paroxysmal positional vertigo (BPPV) was not diagnosed in these subjects.

### Caloric Test and vHIT

Of the nine patients, abnormal CP-value was observed in 77.8% (7/9) of the patients (three patients had bilateral caloric weakness, whereas four patients had unilateral caloric weakness), and 22.2% (2/9) patients had normal caloric result.

Of the nine patients, eight patients (one unilateral and seven bilateral cases) had normal bilateral vHIT results, and one patient (case No. 7) had unilateral pathological vHIT results of three SCCs in the left side (shown in Table 1). Therefore, two patients (2/9, 22.2%) had concordant normal caloric-vHIT results, one patient (1/9, 11.1%) concordant abnormal results, and six patients (6/9, 66.7%) abnormal caloric responses with normal horizontal vHIT results.

## Discussion

### Diversity of Vestibular Manifestations in EVA Patients With Vertigo

This study has shown the diversity of vestibular manifestations in EVA patients with vertigo, which has clinical implications for the differential diagnosis of vertigo.

In our series, two children with bilateral EVA (cases No. 2 and 5) manifested as the post-traumatic vertigo, and both patients showed bilateral caloric weakness and normal vHIT results. By examining the vestibular function of EVA patients with and without vestibular signs, Zalewski et al. (18) found that a history of head injury was associated with both the number of vestibular signs and symptoms and abnormal videonystagmography findings. Although head injury can be a predisposing factor for hearing loss in EVA patient, Zalewski et al. (18) suggested that it cannot infer a causal relationship between head trauma and vestibular dysfunction in EVA patients, as patients with vestibular dysfunction may also be more likely to fall and suffer head injury. Our study found normal vHIT results in these two EVA patients with post-traumatic vertigo. Since vHIT gain is associated with balance and fall risk (19), therefore, we are led to believe that, for the EVA patients with normal vHIT gain, head injury may be associated with the reduced caloric responses. A possible explanation may be that head trauma promotes cerebrospinal fluid reflux and exacerbates endolymphatic hydrops in EVA patients (20, 21), but such an injury may not impair type I vestibular hair cells directly.

In this study, one patient with unilateral EVA with vertigo (case No. 1) showed delayed onset of vertigo, which were similar to those with ipsilateral delayed endolymphatic hydrops (DEH) (22). For this patient with unilateral EVA, because of normal hearing in the non-affected ear, the affected ear was not diagnosed as EVA until imaging was performed after the onset of delayed vertigo. Our findings are in agreement with those of Oh et al. (23), who had first reported a case of EVA with hearing loss since early childhood, but vestibular symptoms could be delayed into adulthood. Song et al. (24) also observed a tendency of later onset of vestibular symptoms compared to auditory symptoms. It is suggested that vestibular system is more resistant than the cochlear system to mechanical or chemical insult, but the mechanism underlying this differential sensitivity of cochlear and vestibular end organ needs further investigation.

In our group, case No. 7 had a sudden deterioration of hearing in one ear with pre-existing hearing loss accompanied by vertigo. The symptoms resembled those of idiopathic sudden deafness accompanied by vertigo, and the results of caloric test and vHIT of the ear with sudden hearing loss were consistently abnormal. These results suggested an acute peripheral vestibulopathy, which damaged both type I and II vestibular hair cells. The possible mechanisms may involve the reflux of hyperosmotic fluid or osmotic and chemical imbalance in endolymph, which may increase the degeneration of vestibular hair cells and lead to vestibular dysfunction (25, 26). In addition, instability of the receptor cell membrane caused by defective chloride iodine channels may also result in this vestibular impairment (23).

In this study, the possibility of comorbid BPPV was excluded by the absence of positional vertigo and negative results of positional tests. In addition to the clinical manifestations of vertigo described above, EVA patients have also been reported to have other types of vertigo, such as BPPV. Song et al. (27) studied the clinical symptoms of five EVA patients with BPPV and speculated the possible pathogenesis, which may be related to pressure reflux or/and hyperosmolar fluid reflux through EVA, which may directly dislodge otoliths from the utricle or accelerate the degeneration of the otolithic membrane, resulting in secondary BPPV. In this study, no EVA patients presented with BPPV, which may be due to the small sample size.

### Characteristics of Angular VOR Test in EVA Patients With Vertigo

In our series, 66.7% of EVA patients showed caloric-vHIT dissociation, that is, reduced caloric response plus normal vHIT result. At present, few reports have focused on these two tests in EVA patients with vertigo. Jung et al. (15) compared the caloric-vHIT function in patients with EVA, MD, and vestibular neuritis. The results showed that 40% of EVA patients had unilateral caloric weakness, and 30% had recurrent vertigo. All patients with recurrent vertigo had positive CP results, but only one patient showed abnormal vHIT result. This inconsistency between the caloric test and vHIT was also exhibited in patients with MD, but not in patients with vestibular neuritis. Besides EVA and MD, disagreement of caloric and vHIT results has been described in many other vestibular disorders, such as DEH and inner ear malformation, which share a common imaging feature, i.e., endolymphatic hydrops (9, 15, 28, 29). Gürkov termed the diseases as hydropic ear disease since endolymphatic hydrops has been morphologically confirmed by inner ear magnetic resonance imaging (MRI) (30). Two hypotheses have been brought up to explain this dissociation: (1) hydrostatic model: the increased diameter of the semicircular duct in hydropic labyrinths resulting in a smaller thermally induced pressure across the cupula, while the increased duct diameter has little effect on responses to rotation (28). (2) “dual frequency” of vestibular hair cells: it is believed that type II hair cells are sensitive to the low-frequency (caloric) stimulus and the type I hair cells to the high-frequency (head impulse) stimulus. Previous histological studies addressing the damage pattern of vestibular hair cells in patients with MD have yielded controversial findings (8, 31). However, selective damage of vestibular hair cells in patients with EVA has been rarely reported. Currently, several studies, including pathological and imaging (inner ear gadolinium MRI) studies (24, 32), indicate the presence of endolymphatic hydrops in patients with EVA. The inconsistency of caloric-vHIT in our series also support the theory that caloric-vHIT dissociation might be a common pattern of vestibular deficit in hydropic ear disease. Moreover, the degree of unilateral caloric weakness should be considered when interpretating the correlation of caloric-vHIT results. Since vHIT is a low-sensitivity, high-specificity test for detecting horizontal VOR pathology compared to the caloric test, horizontal vHIT responses are commonly normal until caloric weakness is very severe (33, 34). This is compatible with our findings, as only patient No. 7 with severely reduced unilateral caloric reflex (CP-value = 74%) exhibited an abnormal vHIT response.

In the present study, the incidence of caloric-vHIT inconsistency (66.7%) was higher than that reported by Jung et al. (40%) (15). The reason could be that only patients with vestibular symptoms were included in our study. In the study by Jung et al. (15), three patients with EVA had dizziness, all of them showed unilateral caloric weakness and one case also had positive vHIT. The incidence of their caloric-vHIT dissociation was two-third, which is in agreement with our results.

In this series, there were eight patients with bilateral EVA. Three of them had bilateral caloric hypofunction, and four showed unilateral CP. The reason for bilateral EVA while exhibiting unilaterally decreased caloric responses is unknown. Zhou et al. (14) reported that 14 of 16 patients with bilateral EVA who received caloric tests had vestibular hypofunction (12 bilateral, two unilateral). In a cohort of 22 patients with EVA (including 19 with bilateral lesions), eight patients underwent caloric tests. Four of them had unilateral vestibular hypofunction and two of them had bilateral vestibular hypofunction (24). The finding of unilateral hypofunction to caloric reflex in bilateral EVA patients is inconsistent with the results of animal studies using pendrin knockout mice, which exhibit vestibular signs behaviorally and bilateral Pendred syndrome-associated vestibular lesions histologically (26). Jung et al. explained that environmental factors (such as head trauma) might be responsible for the development of vestibulopathy instead of genetic variation (15). Interestingly, the only patient with unilateral EVA in our series did not exhibit unilateral caloric hypofunction. Environmental factors or the fluctuation of caloric response might account for this (21). Therefore, the angular VOR function in unilateral and bilateral EVA patients should be monitored dynamically in future studies.

Due to the relatively small sample size, our study did not analyze the relationship between the audio-vestibular function and the severity of the vestibular aqueduct enlargement. Previous studies have drawn inconsistent conclusions on this relationship. Yetiser et al. (35) found no correlation between the level of electronystagmographic abnormality and the severity of radiological deformity in a series of 10 EVA patients. Ishida et al. (36) studied nine patients with EVA and found that the morphology of SCCs was associated with vertigo, the large lateral SCC fluid-containing ratio was closely associated with the presence of vertigo, but the endolymphatic duct and sac volume was not related to the pathophysiology of vestibular function. In pediatric EVA patients, midpoint and operculum size were correlated with cervical VEMP and caloric response, greater high-frequency PTA was positively correlated with unilateral weakness (37). This may be explained by the proximity of the basilar turn of the cochlea and the vestibular apparatus. Therefore, inconsistencies in these previous findings may be due to small sample sizes and large individual variations. Also, the fluctuating vestibular function should be considered (21).

The main limitation of this study is the small sample size. The reason is that most EVA patients are referred to our clinic for hearing loss rather than for vertigo and vestibular complaints are relatively rare in EVA patients compared to audiological ones. Furthermore, due to ethical considerations, laboratory vestibular tests were not routinely performed in EVA patients (especially for the pediatric patients) without vestibular symptoms in this series. Another limitation was that the imaging criteria employed to diagnose EVA in this study were the classic Valvassori criterion. Compared to the recent Cincinnati criteria, the lower sensitivity of Valvassori criterion may also contribute to the small sample size involved (38). Therefore, the small sample size may limit the generalizability of our findings and future prospective study involving a larger cohort of EVA patients are warranted.

## Conclusions

In addition to hearing loss, patients with EVA may also exhibit a broad clinical spectrum of vestibular symptoms. Our study showed that EVA patients with vertigo can present with a reduced caloric response and normal horizontal vHIT, and this pattern of angular VOR impairment was also found in other hydropic ear diseases.

## Data Availability Statement

The original contributions presented in the study are included in the article/supplementary material, further inquiries can be directed to the corresponding author/s.

## Ethics Statement

The studies involving human participants were reviewed and approved by The Ethical Committee of Union Hospital Affiliated to Tongji Medical College of Huazhong University of Science and Technology, Wuhan, China. Written informed consent to participate in this study was provided by the participants' legal guardian/next of kin.

## Author Contributions

BL: study conception and design, data acquisition, and critical review of the manuscript. YL: data analysis and interpretation, drafting, and revision of the manuscript. ML: drafting and revision of the manuscript. All authors contributed to the article and approved the submitted version.

## Funding

This work was supported by grants from the National Natural Science Foundation of China (NSFC NO. 81670930), the Natural Science Foundation of Hubei Province, China (No. 2016CFB645), and Fundamental Research Funds for the Central Universities, China (No. 2016YXMS240).

## Conflict of Interest

The authors declare that the research was conducted in the absence of any commercial or financial relationships that could be construed as a potential conflict of interest.

## Publisher's Note

All claims expressed in this article are solely those of the authors and do not necessarily represent those of their affiliated organizations, or those of the publisher, the editors and the reviewers. Any product that may be evaluated in this article, or claim that may be made by its manufacturer, is not guaranteed or endorsed by the publisher.
